# Can Digital Tools Fix Bias in Mental Health Triage?

**DOI:** 10.2196/100947

**Published:** 2026-05-28

**Authors:** Beth Rush

**Keywords:** mental health, digital technologies, internet, mobile applications, telemedicine, artificial intelligence, algorithmic bias, bias, health equity, clinical decision support systems, human factors, human-in-the-loop

## Abstract

Recent advances in digital technologies hold both promise and potential pitfalls for mental health triage. In this *News and Perspectives* article, JMIR Correspondent Beth Rush reports on the issue of bias and the role of digital tools in the mental health context.

**Key Takeaways:**

- Digital tools can create standardization and transparency in mental health triage, but they can also inherit and even amplify bias.
- The most effective solution is a hybrid model that combines technology with human expertise.

The growing intersection of mental health and digital technologies is increasingly positioned as a solution to one of health care’s most persistent barriers to health equity—biased triage.

Health systems under pressure are adopting algorithmic tools to improve consistency, reduce subjectivity, and scale access. The logic is compelling. Structured systems should, in theory, produce more equitable outcomes than human judgment alone. Yet, the reality is more complicated: while digital technologies offer meaningful advantages for mental health, they also reflect the very inequities they aim to resolve.

## The Bias Problem

Bias in discussions of mental health and digital technologies often begins with the human side of the equation. Clinical triage is conducted in environments defined by time constraints, incomplete information, and high stakes. Under these conditions, decision-making frequently relies on heuristics, which can introduce conscious and unconscious biases.

A 2026 study conducted by researchers at Ann & Robert H. Lurie Children’s Hospital of Chicago provides a clear example. In its analysis of over 74,000 pediatric emergency department visits, researchers found that undertriage—assigning a lower severity score than the level of care a child actually needed—was more likely for children who were Black or Hispanic or those who preferred Spanish over English.

Jennifer Hoffmann, MD—the study’s lead author—explains that these disparities have multiple drivers. “The disparities we observed may be driven by a combination of implicit bias, communication barriers, and structural factors within the healthcare system,” she says. “For example, clinicians may unconsciously perceive symptoms differently based on race or ethnicity, and underuse of professional interpreters can lead to miscommunication for Spanish-speaking families. These factors, along with broader systemic inequities, can influence how urgency is assessed during triage.”

Empirical research has repeatedly shown disparities in how patients are assessed and treated. Race, gender, and socioeconomic status influence diagnostic outcomes, access to care, and perceived risk levels. These patterns are rooted in long-standing structural inequalities that shape the entire mental health ecosystem. Any attempt to address bias must contend with these systemic realities rather than treat bias as an isolated issue.

**Figure:**
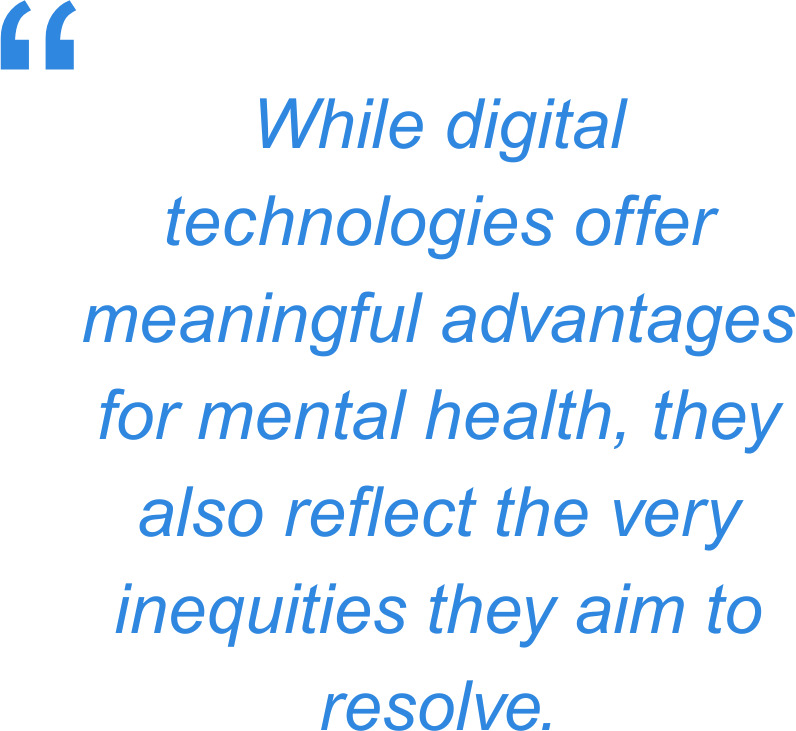


## The Role of Clinical Decision Support

Triage platforms—digital clinical decision support tools designed to standardize and support clinical decision-making—appear promising for mitigating bias. They include artificial intelligence (AI)–driven risk prediction models, structured digital intake platforms, and symptom assessment tools that guide patients and clinicians through consistent workflows. By applying the same criteria across cases, these systems aim to limit the influence of individual judgment. They can prioritize patients based on risk, flag urgent cases, and suggest care pathways, particularly in settings where specialist expertise is limited.

Sam Zand, MD—a psychiatrist and CEO of Anywhere Clinic—notes the complexity of mental health triage. “When evaluating mental health patients in the emergency department, it can often be difficult to assess the level of risk related to care of these patients,” he explains. “Misclassification of risk will lead to either an under-triaged patient and/or over-triaged patient—both of which have dire consequences regarding patient safety and staff resources.”

Kenneth Perry, MD—an emergency physician in South Carolina—sees potential for AI to improve these challenging decisions. “AI tools would be very helpful in determining which patients need inpatient medical care and which are safe enough for outpatient follow up,” he says. “If there was a way to keep the bias of the questioner from the interaction, more correct, efficient dispositions could be possible.”

## The Challenge of Algorithmic Bias

Despite their potential, however, the effectiveness of these tools is fundamentally constrained by data quality. These systems are trained on historical datasets that often reflect the very disparities found in the Ann & Robert H. Lurie Children’s Hospital of Chicago study.

Mai Uchida, MD—a pediatric psychiatrist and AI investigator at Harvard University and the NTT Research Brain Science Center, and director of pediatric depression at Massachusetts General Hospital—explains how bias becomes embedded in these systems. “If historical data reflects disparities—such as underdiagnosis of internalizing disorders in boys, overdiagnosis of behavioral disorders in Black children, or differential access to care across socioeconomic groups—then AI may not only reproduce these patterns but reinforce them with a veneer of objectivity,” she says.

Flawed assumptions can get amplified. According to Dr Saravanan Thirumalai Thangarajan, visiting scientist at Harvard T.H. Chan School of Public Health and international fellow at Ariadne Labs, Brigham and Women’s Hospital, “AI triage tools in pediatric mental health do not neutralize bias. They can industrialize it. Many triage models use prior healthcare utilization as a proxy for need, but utilization reflects access, not illness burden. A model trained on unequal access patterns does not simply detect risk. It learns who the system already knows how to see.”

**Figure:**
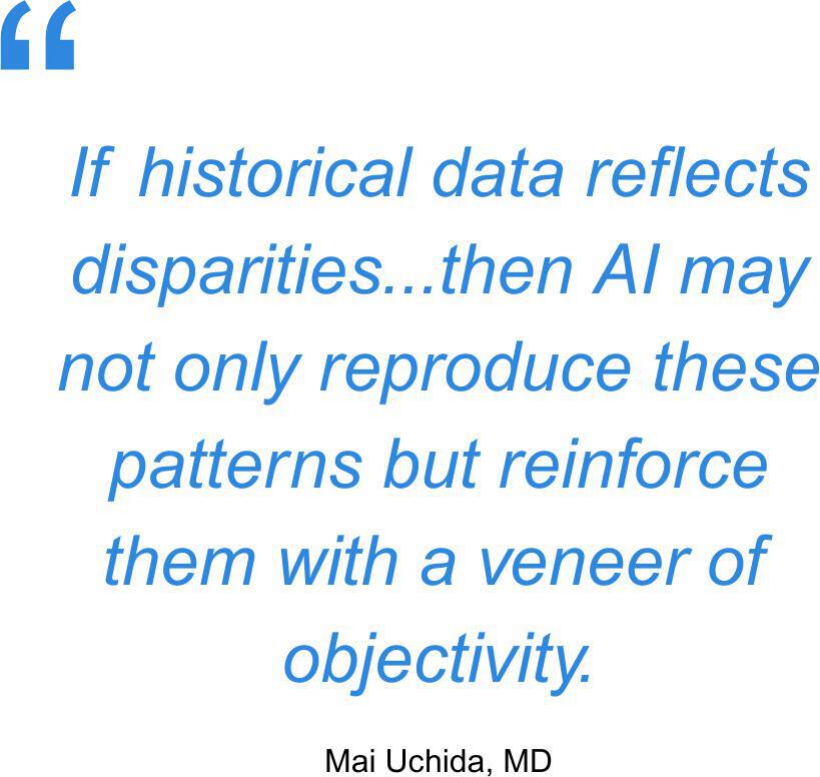


## The Value of Standardization and Transparency

Yet, these tools have practical value that shouldn’t be dismissed. When designed and implemented carefully, they can reduce certain types of bias and strengthen triage systems.

One of their most important contributions is standardization. Digital intake tools ensure that every patient is asked the same core questions consistently. This reduces variability in data collection and limits the influence of interpersonal dynamics during initial assessments.

Transparency is another key advantage. Algorithmic systems can be stress-tested and calibrated, and decisions made with digital systems can be analyzed at scale, enabling the detection of patterns of disparity that might otherwise be difficult to identify in traditional settings. This allows organizations to audit performance, iteratively refine models, and address bias more systemically.

Corrective measures can also be explicitly and proactively incorporated into digital triage tools. Marie Deschamps, PhD, LPC-A, ATR-P—a clinician-scientist, digital health innovator, and visiting scholar at The New School—emphasizes the importance of capturing diverse forms of patient expression. “Integrating multimodal data streams—including images, voice, and narrative—into AI-supported triage systems offers a critical pathway to reduce bias in mental health care by honoring culturally diverse, non-verbal, and metaphor-based expressions of distress rather than forcing them into standardized, and often inequitable, clinical categories,” she explains.

## Toward a Human-in-the-Loop Model

Digital tools cannot fully eliminate bias in mental health triage. The issue is too deeply embedded in broader social and institutional structures.

Mental health triage often involves ambiguity, requiring contextual understanding and ethical judgment. These are areas where human expertise is indispensable. At the same time, it also requires consistency and standardization, which is precisely why digital support systems can be valuable.

The most effective approach, then, is a hybrid, or human-in-the-loop, model. In this framework, digital triage tools provide standardized inputs and analytical support, while clinicians apply judgment and context. This balance allows for consistency and adaptability in decision-making.

Hoffmann points to concrete steps that can be implemented immediately. “I think there are several practical steps emergency departments can take right away, including improving access to and use of professional interpreters during triage and standardizing how interpreter needs are identified,” she says. “Even small workflow changes can make a meaningful difference in promoting more equitable triage decisions.”

